# Supplementary material for: Are carbon emissions trading and green financial instruments synergistic? -Comprehensive quantitative research based on content analysis
Source: PLoS One. 2024 Mar 7;19(3):e0298601. doi: 10.1371/journal.pone.0298601 (PMC10919601; doi:10.1371/journal.pone.0298601)
Supplement: S1 Data — (ZIP) [file pone.0298601.s001.zip › Data/地级市Z_F工作报告.zip!/╔╜╢1⁄2/▒⌡╓▌╩╨/└┤╫╘╣1⁄2╓┌║┼í╛┬φ┐╦╩2╛▌═°í┐_2006.docx]

各位代表:

　　现在,我代表市人民政府,向大会作政府工作报告,请予审议,并请市政协委员和其他列席会议的同志提出意见。

　　一、解放思想,干事创业，“十五”时期经济社会发展实现新跨越

　　“十五”时期,是我市发展史上最快最好的时期之一。在省委、省政府和市委的坚强领导下,在历届地市领导班子打下的良好基础上,市政府团结依靠全市人民,高举邓小平理论和“三个代表”重要思想伟大旗帜,认真贯彻落实党的十六大和十六届三中、四中、五中全会精神,以科学发展观统领全局,按照“坚持‘五个统筹’,建设系统‘九州’”的总体部署,坚持“发展是主题,调整是主线,招商是重点,实干是关键,稳定是基础,廉政是保证”的工作方针,认真执行市人大及其常委会的各项决议,聚精会神搞建设,一心一意谋发展,经济社会发展进入了新的发展阶段。

　　(一)综合经济实力迅速增强。2005年实现生产总值667.27亿元,“十五”期间,年均增长15.5%,比“九五”时期高2.7个百分点;人均生产总值实现2201美元。地方财政收入达到32.1亿元,在全省前移2个位次,按可比口径年均增长37.36%。辖区内本外币存款余额406.22亿元,贷款余额352.33亿元,年均分别增长15.9%和23.2%。三次产业结构不断优化,由2000年的22.7:48.3:29调整到2005年的13.3:60.2:26.5。

　　(二)工业化进程明显加快。制定实施了“1238”工程,超前谋划十大产业链,“工业兴市”战略全面推进,传统优势产业巩固发展,飞机、汽车、轮船及零部件制造等新兴产业开始起步。五年内全市工业总投入达到430亿元,其中魏棉、鲁北、滨化、活塞、京博、亚光等13家大企业领办工业园累计完成投资200亿元。2005年全市规模以上工业实现增加值304.85亿元,年均增长28.38%,利润、利税分别达到65.63亿元和114.79亿元,比2000年分别增长4.03倍和4.22倍。高新技术产业产值达到96.78亿元,年均增长114.59%。

　　(三)“三农”工作扎实推进。农业综合生产能力稳步提高,农民收入持续增长。2005年农林牧渔业总产值168.85亿元,农民人均纯收入3817元,年均分别增长4.3%和9.23%。冬枣、畜牧、蔬菜等“五大带动农户工程”成效显著,农业标准化、产业化水平不断提高,标准化基地达到144万亩。农村“水电路医学”等基础设施明显改善,五年新增蓄水能力3.7亿立方米,解决了129.2万农村人口的吃水问题。农村税费改革稳步推进,累计减轻农民负担3.93亿元,人均减负131元。

　　(四)城市基础设施建设实现重大突破。五年全社会固定资产累计投资1169亿元,年均增长45.7%。“四环五海”工程基本完工。组团式、连带型城市群框架初步拉开。各县区总体规划基本完成,各县城和中心镇面貌发生显著变化,城镇功能显著提高。滨州新区、经济开发区建设顺利推进。相继建成了小开河引黄灌区续建配套工程、滨博及滨大高速公路、黄河二桥、国道220线一级路改造等一批重大基础设施项目;开工建设了滨州铁路滨港线及黄河公铁大桥、威乌和京滨高速滨州段、滨州港经济园区、大高航空城、城区“两河”治理等重点项目。五年新增高速公路46公里,新建、改造一级公路130公里、城市道路333公里;每百人拥有电话52.8部。新兴服务业迅猛发展,旅游产业实现新突破,五年接待国内游客增幅和旅游总收入增幅均列全省前茅。

　　(五)对外开放成效显著。相继开展了“招商引资年”、“招商引资优惠年”、“加大投入年”活动,五年实际利用境外资金累计10.55亿美元,年均增长67%。2005年进出口贸易额达到22.44亿美元,年均增长43.7%。华纺股份、魏桥纺织、滨州活塞、西王糖业四家企业相继在境内外上市,上市公司达到5家,总融资58亿元人民币。成功举办了四次银企合作活动,签约资金1067.61亿元,前三届实际到位资金563.4亿元,平均资金到位率达到95.1%。

　　(六)区域经济趋向协调发展。认真落实县域经济发展和“对口帮扶”政策,大力实施“渡河战役”,促强、扶弱、带中间,20个强乡镇带动作用显著增强,20个弱乡镇落后面貌逐步改变,县域经济综合实力跃上新水平,黄河南北县区协调发展呈现新格局。高起点谋划“北带”开发,共建成防潮大堤158公里,护砌22.8公里,滨海经济开始起步,沿海生态科技产业开发取得阶段性成果。

　　(七)社会事业全面进步。深入实施“科教兴滨”战略,五年引进科技成果1091项,引进科技人才2969人。“普九”教育水平进一步提高,“以县为主”农村义务教育管理体制不断完善,农村校舍改造走在全省前列,建成了滨州学院、职业学院、高级技校,人均高教资源跻身全省前列。公共卫生两个体系建设任务全面完成,农村新型合作医疗试点工作稳步推进。人口与计划生育综合改革继续深化,“十五”人口计划圆满完成。文化体育事业进一步繁荣,群众性文体活动日趋活跃,成功举办了一系列国际性、全国性节会赛事,竞技体育成果实现了历史性突破。国防建设、双拥共建、广播电视、外事侨务、对台事务、民族宗教、人防、地震、气象、档案、地名、史志、新闻、出版、统计、物价、职工教育、油区管理等工作不断加强,妇女、儿童、老龄和残疾人事业得到重视和发展。

　　(八)人民生活和社会保障水平稳步提高。2005年城镇居民人均可支配收入10286元,五年年均增长10.3%。社会保障覆盖面进一步扩大,“两个确保”得到巩固,城乡低保实现应保尽保;五年新增城镇就业再就业17万人,城镇登记失业率控制在4%以内,全市参加基本养老保险人数达到25.5万人,离退休人员社会化管理服务率达到98%,社区服务率达到76%。深入推进“六河水变清”工程,规划建设了9个污水处理厂,资源节约型和环境友好型社会、生态市建设及国家环保模范城市创建活动开始启动。全市森林覆盖率达到21.3%,比“十五”初增长8.8个百分点,空气质量良好率达到97.53%。

　　(九)政府行政能力进一步强化。全面推进“服务政府、责任政府、阳光政府、法治政府、高效政府、诚信政府”建设,政府效能效率明显提高。沉着应对非典、禽流感等重大疫情和特大风暴潮等自然灾害,政府应急管理能力进一步增强。着力解决了土地征用、城镇房屋拆迁、违法排污、企业重组改制和破产中损害职工利益及拖欠农民工工资等问题,依法行政水平不断提高。加快体制机制创新,全面推行了行政审批制度、国有资产监管体制、财政体制等各项改革。依法治市全面推进,监察、审计等行政监督不断加强,安全生产形势保持稳定,“平安滨州”建设取得了明显成效。精神文明、民主法制、廉政建设取得新成效,全市稳定和谐局面得到巩固和发展。

　　各位代表,“十五”时期,我市经济建设和社会事业取得了巨大成就,为今后的跨越发展奠定了坚实基础。今天的滨州,已经以崭新的形象站在了一个新的历史起点上!同时,我们也清醒地认识到,我市经济总量少、基数小,欠发达、欠发展的状况还没有根本改变;与先进市的差距及黄河南北的差距还比较大;经济结构不合理,三产明显滞后,自主创新能力不够强,粗放型增长方式还没有根本转变;农业综合生产能力仍然较弱,农民增收的长效机制还没有建立完善起来;高层次人才和技能型人才不足;保障体系、救助体系建设及环境保护等工作任重道远;社会稳定和安全生产方面还有许多亟待解决的问题;政府职能转变还不够到位,投资软环境仍不尽人意,一些地方、单位和政府工作人员脱离群众、作风浮躁、服务意识淡薄;少数干部还存在侵犯群众利益的行为,极少数人以权谋私、腐化堕落;就医难、上学难等一些关系群众切身利益的问题还没有解决好。对这些问题,我们一定高度重视,认真对待,采取得力措施,切实加以解决。

　　二、扎实苦干,拼搏决战，2005年重点工作任务圆满完成

　　2005年,我们按照“干劲一鼓再鼓,决战2005”的总体要求,奋发进取,较好地完成了市八届人大三次会议确定的各项任务。

　　经济工作六大重点强势推进。(一)立足工业兴市,全力实施“1238”工程。加大投入,优化结构,着力提高工业经济运行质量。2005年魏桥创业集团、滨化集团销售收入突破350亿元和100亿元,分别列中国500强企业排行榜109位和239位;渤海油脂和京博集团分别入选中国制造业企业500强和中国服务业企业500强;年销售收入30-100亿元的企业达到3家;10-30亿元的企业达到13家;规模以上企业达到997家。新增中国名牌1个,山东名牌12个。(二)外经外贸、民营经济、高新技术产业“三个亮点”实现新突破。全市出口总额12.41亿美元,增长37.3%。民营经济实缴税金24.98亿元,增长30.6%。高新技术产业发展步伐加快,占规模以上工业总产值的比重达到8.06%。实现社会消费品零售总额179.73亿元,增长15.6%。(三)扎实开展“三农工作年”活动。积极推进农村经济结构调整,新增省重点龙头企业12家,达到22家,新增市重点龙头企业39家,达到97家,辐射带动农户47.7万户;全面落实各项惠农政策,全年农民人均减负38元;我市被正式命名为全国水土保持生态环境示范城市。(四)加快基础设施和城市建设。实施建设了331个重大基础设施项目,全年完成投资144亿元,比上年增长45.5%,铁路、港口、高速公路建设全面推进,滨大高速、西外环、北外环、220国道一级路改造顺利通车。新区建设和旧城改造取得新进展,新增城市道路150万平方米,城市绿化面积80多公顷,各类建筑面积154万平方米。(五)招商引资和县域经济发展取得重大突破。全年引进市外资金500.97亿元,其中实际利用境外资金2.36亿美元,分别增长45.25%、30.9%;强化重点帮扶工作,促进南北协调发展,各县区比学赶超,亮点纷呈,邹平县进入“全国双百强”,在全国县域经济基本竞争力百强县中列第62位,无棣等三个欠发达县均呈现良好发展势头。(六)各项改革全面深化。市属国有工业企业改革改制任务基本完成;培植上市后备资源企业20家,西王糖业在香港红筹股上市,融资6.1亿港元;成功举办了第四次银企合作推进会,签订合同、协议项目599个,贷款金额478.32亿元,比上年增长70.75%。

　　社会发展六项工作成效显著。(一)加大科技教育事业投入。省级规范化学校达到53所,普通高中招生万人比达到195人,跃居全省中游水平;全年申请专利271项,新上科技示范项目136项,引进科技人才361人、科技成果231项。(二)落实就业再就业各项政策。全市新增城镇就业再就业4.8万人,其中下岗失业人员再就业1.3万人;新增劳务输出7.27万人。社会保险扩面征缴迈出新步伐。(三)统筹发展各项社会事业。市“三馆”和奥林匹克公园初步完成方案设计,成功举办了第三届体育节,我市运动员在十运会和一系列国际大赛中取得好成绩。公共卫生两个体系建设16个项目全面完工,邹平县成为全省第一个国家级卫生县城。低生育水平保持稳定,人口自然增长率5.32‰。(四)民主法制建设进一步加强。各级政府及部门自觉接受人大及其常委会的依法监督和政协的民主监督,充分发挥民主党派和人民团体的作用,广泛听取人民群众的批评和建议。全面完成了“四五”普法的各项任务。深入开展投资环境综合整治,加强了效能效率监察和各项审计工作。(五)积极开展“和谐稳定年”活动。组织了每月大接访和民主议政日活动,较好地处置了社会转型期的各种矛盾和问题。(六)大力弘扬“奋斗、和谐、超越”的滨州精神。扎实开展文明创建活动,平安滨州、文明滨州、和谐滨州建设取得明显成效。

　　八项民心工程圆满完成。(一)全年新增蓄水能力8100万方,解决和改善了30.1万人的吃水问题。村村通自来水率达到66%,全省现场会议在滨州召开。(二)邹平县和滨城区作为省级农村新型合作医疗试点,参合率分别达到86.9%和83.08%;博兴县试点乡镇参合率达85%;年内计划改造乡镇卫生院14家,完工13家,已有88%的乡镇、86%的行政村卫生室实现了乡村卫生一体化管理。(三)农村公路改造,开工建设2653公里,基本实现了村村通油路、通客车。(四)全市有线电视通村率达86.2%,博兴、邹平、惠民、滨城、开发区等五个县区基本实现了村村通,通户率达84.7%。(五)对秦台干沟实施疏浚护坡、管网配套、雨污分流、绿化美化四位一体综合整治,到今年5月,秦台干沟将彻底告别严重污染的历史,一条9.7公里长的水清树绿的城市景观带将展现在全市人民面前。13.9公里的新立河治理及配套工程全面完成,黄河八路的露天污水得到根本性治理。(六)建设和改造城市道路66条,总长度达191公里,是历年来投入规模和工程量最大的一年。(七)经济适用住房项目开工建设。(八)建立完善了十项城乡“低保”制度,有5.2万城乡低收入人口实现了应保尽保,对城市低保对象发放了供暖补贴,对低于城市住房标准的困难户和无房户发放了住房补贴;改革农村五保供养体制,提高供养水平,全市集中供养率达到41%。

　　政府自身建设八件实事全面落实。(一)市公共行政审批服务中心去年4月18日正式运行,790项审批服务事项全部纳入中心受理,全年共受理并办结审批项目2.2万余件,符合条件的审批事项受理率100%,按时办结率100%,投诉率为零,提高了办事效率,方便了人民群众。(二)市长公开电话受理中心共受理群众来电4.17万件,办结率99.9%,反馈率99.9%,满意率99.7%,初步架起了政府和人民群众的连心桥。(三)完成政府采购额5.29亿元,节支率达11.93%,其中市级政府采购额2.92亿元,节支率达12.56%。(四)市级电子政务平台投入使用,政府门户网站已经试开通。(五)制定实施了《滨州市行政首长问责暂行办法》、《滨州市国家行政机关及其公务员行政不作为问责办法》,有效地规范了行政行为。(六)建立了政务督查工作规程,全方位强化了督查督办工作,保证了市委、市政府重大决策和重大事项的落实。(七)实施了重大决策咨询听证制度,凡涉及经济社会发展的重大决策和关系群众切身利益的重大事项,都邀请人大代表、政协委员、基层群众列席市政府常务会议或全体会议,吸收高校和科研机构的专家学者咨询论证。(八)《滨州市人民政府公报》2005年1月1日正式创刊,每月一期免费发放到村、单位和人大代表、政协委员,增强了政府工作透明度,促进了阳光政府建设。

　　各位代表,回顾五年特别是2005年的工作历程,我们深深感到,政府工作取得的每一点成绩,都得益于党中央、省委、省政府的英明决策,得益于中共滨州市委的正确领导,得益于人大、政协和社会各界的大力支持,得益于全市人民的艰苦奋斗和无私奉献,凝聚着全市人民的智慧和心血。在此,我代表市人民政府,向全市广大工人、农民、知识分子、干部职工、人民解放军驻滨部队、武警官兵,向各民主党派、工商联、无党派人士和社会团体,向关心和支持滨州建设的中央、省驻滨单位,向所有关心和支持我市发展的港澳台同胞、海外侨胞和海内外朋友,表示崇高的敬意和衷心的感谢!

　　三、同心同德,奋力跨越，推进“十一五”时期经济社会发展又快又好

　　各位代表,“十一五”时期是滨州全面建设小康社会的关键时期。按照中央、省委、省政府和市委的部署,市政府认真学习贯彻党的十六届五中全会精神,深入调查研究、广泛征求意见,编制了《滨州市国民经济和社会发展第十一个五年规划纲要(草案)》,业经中共滨州市委六届十次全委会审议。《纲要(草案)》及《说明》已印发大会,请一并予以审议。

　　市政府在编制《纲要(草案)》时,注意突出规划的前瞻性、全面性、客观性、特色性和可行性,突出滨州区域发展与国家、省经济社会发展的方向性趋势相一致,经济发展与社会各项事业发展相统一,总体规划与专项、专业规划相结合。综观“十一五”时期的国内外环境,总体上看机遇与挑战并存,机遇大于挑战。我们要坚持以邓小平理论和“三个代表”重要思想为指导,全面贯彻落实科学发展观,紧紧抓住本世纪头二十年的重要战略机遇,以跨入全省先进行列为目标,进一步增强自主创新能力和体制机制活力“两大动力”,加快实施工业兴市、城市带动、人才强市“三大战略”,突出打造十大产业(链)集群、招商引资、统筹城乡发展、建设节约型友好型社会“四大重点”,全力建设系统“九州”,构建社会主义和谐社会,力争提前全面建成小康社会。经济社会发展的战略定位和布局是:南融山东半岛城市群和济南都市圈,北接天津滨海新区,面向环渤海,服务京津济,努力打造黄河三角洲中心城市、全国棉纺家纺之都、现代化生态园林旅游城市,突出“中心”(滨州市区),强化“龙头”(邹平县),培植“两带”(博滨沾经济带和惠阳无经济带),形成良性互动的战略发展格局。综合考虑发展阶段、发展环境和基础条件,《纲要(草案)》从经济繁荣、结构合理、社会和谐、生活富裕、环境优美五个方面,提出了“十一五”期间的发展目标。在效益提高、结构改善、消耗降低的基础上,全市生产总值达到1400亿元,年均增长15%左右,人均达到4500美元;三次产业结构调整为8.5:60:31.5;地方财政收入超过98亿元,力争达到百亿元,年均增长25%以上;单位生产总值能源消耗每年降低4.5%。工作上重点把握好以下八个方面:

　　(一)加快建设社会主义新农村,促进“三农”工作迈入新境界。

　　按照“生产发展、生活宽裕、乡风文明、村容整洁、管理民主”的总体要求,紧紧抓住增加农民收入这个重点,坚持以工带农、以城促乡、工业反哺农业、城市支持农村。加大水利建设投入,调整优化农业结构,着力建设北部沿海水产品养殖区、中北部优质林果及畜牧业养殖区、中部城郊农业区、沿黄优质粮棉和水产畜牧养殖区、南部花卉和林果区等五大特色农业经济区。建立农产品质量安全体系和检疫检测体系,推进农业产业化、标准化。加大农村公共事业投入,尽快改善广大农村的生产生活条件和整体面貌。

　　(二)加快推进结构调整和增长方式转变,构筑产业发展新体系。继续实施工业兴市战略,以“1238”工程为总抓手,巩固提升优势产业、做大做强优势企业,引进开拓新兴产业、做高做好新兴企业,全力打造十大产业链。到2010年,规模以上工业增加值达到760亿元,年均增长20%以上。对66家优势骨干企业全方位支持倾斜,魏桥创业集团销售收入力争过1000亿元。着力提高服务业占生产总值的比重,加快完善公路、铁路、港口等基础设施,推动电源基地建设。配合国家南水北调水利工程,实现水资源的综合利用和优化配置。

　　(三)加快实施城市带动、南北联动,形成区域协调发展新格局。按照“城市带动、城乡统筹、南北联动、协调发展”的思路,完善城市功能,提高城市品位,实施好新一轮城市总体规划,加快建设组团式、连带型城市群。加大对口帮扶力度,全力突破阳信、惠民,促进北部地区快速崛起。抓住黄河三角洲高效生态经济区建设的机遇,大力发展高效生态经济和绿色产业。加快“北带”开发进程,培植富有活力的经济隆起带。

　　(四)加快提高自主创新能力,打造经济发展新支撑。积极发展高新技术产业,促进传统产业改造升级。到2010年,力争省级以上高新技术企业发展到130家,高新技术产业产值达到550亿元;拥有自主知识产权高新技术产品的比重达到60%以上。大力推进人才强市战略,抓好人才的引进、培训。完善科技投入机制,加强对知识产权的保护和管理,不断优化创新环境。

　　(五)加快发展繁荣社会事业,实现经济社会协调发展新跨越。

　　巩固普及九年义务教育成果,基本普及高中阶段教育。大力发展职业教育,鼓励发展民办教育,重视发展学前教育,积极发展成人教育和继续教育,加快发展高等教育。完善公共卫生疾病预防控制和医疗救治两个体系,加强乡镇卫生院建设,规范村级卫生机构,全面推行新型农村合作医疗,提高卫生安全应急和保障能力。深化文化管理体制改革,大力发展文化产业,实施广播电视数字化工程。加快体育基础设施建设,开展全民健身运动,提升竞技体育水平。稳定低生育水平,优化人口出生结构,提高人口素质,全面建立完善人口计生工作新机制。

　　(六)加快建立公平保障机制,促进和谐滨州建设进入新阶段。

　　按照中央建设和谐社会提出的“民主法治、公平正义、诚信友爱、充满活力、安定有序、人与自然和谐相处”总要求,建立健全保障人民群众经济、政治和社会利益的机制。努力扩大就业,加强公共就业服务机构建设,引导农村劳动力合理有序流动。完善社会保障机制,进一步扩大覆盖面,健全城乡低保、农村五保和困难群众救助等配套救助体系。完善收入分配制度,调节部分社会成员收入差距过大的问题。加强安全生产和社会稳定工作,全面提升“平安滨州”建设水平。

　　(七)加快节约型社会和生态市建设,实现可持续发展新突破。

　　大力发展循环经济,推广鲁北集团、西王集团的经验,构筑企业、行业、社会三个层面的循环经济体系。积极发展节水型农业,加快城市供水、排水管网更新改造,全面推进节水型社会建设。实施绿色通道、平原绿化、沿海防护林、荒山荒滩等绿化工程,建设与保护海洋生物、滨海滩涂湿地等自然生态系统,力争到2010年,城市建成区绿化覆盖率达到35%,城镇人均公共绿地面积10平方米。

　　(八)加快改革开放步伐,增创经济社会发展新优势。深化国有企业和国有资产管理体制改革,推进公用事业和垄断行业改革。加快行政管理体制和事业单位改革,逐步建立起新型管理体制。深化财税体制改革,完善市县财政管理体制,健全市级财政转移支付制度。进一步推动企业上市,资本证券化率继续走在全省前列。大力推进经济国际化战略,着力提高利用外资规模和水平,提高出口产品档次和附加值,推动加工贸易转型升级。

　　过5年的努力,力争全市综合经济实力进一步增强,经济增长的质量和效益全面提高,生态环境明显改善,人民群众获得更多的实惠和利益,社会更加文明、和谐、进步,充分展现黄河三角洲生态园林型现代滨州的崭新面貌!

　　四、突出重点,全力冲刺，为“十一五”发展创造良好开局

　　2006年是实施“十一五”规划的开局之年,也是滨州实现由“追赶战略”向“赶超战略”转变的冲刺之年。今年的政府工作要按照省委、省政府和市委的总体部署,以科学发展观统领全局,继续执行“30字”工作方针,突出超越发展“一个主题”,冲刺2006,锁定到2007年经济总量进入全省中游、人均指标进入上中游从而彻底改变欠发达面貌“两个目标”,抓住班子实干、招商投入、环境整治“三个关键”,狠抓强势工业、高新技术、现代服务业、循环经济“四个发展”,扎实推进农村城镇化、农业产业化、南北一体化、环境生态化、社会和谐化“五个加快”,加速形成十大产业(链)集群,推进经济社会又快又好发展,为打造系统“九州”、建设现代滨州奠定坚实基础。力争全市GDP增长17.5%以上,达到800亿元;固定资产投资增长30%以上,达到650亿元;地方财政收入增长25%以上,达到40亿元以上,为“十一五”开好头、起好步。按照上述要求,市政府确定今年突出抓好十件大事。

　　(一)扎实推进社会主义新农村建设。把发展农村经济作为中心任务,对“三农”的扶持由主要靠“少取”转向“多予、放活”,全面取消农业税,进一步扩大公共财政覆盖农村范围,让更多的公共服务惠及农民。市级财政安排“三农”方面投入6479万元,增长65.07%。加快畜牧、水产、林果业发展,优化农业经济结构。不断提升农业产业化、标准化水平,年内市级龙头企业达到100家以上,各类农业标准化基地面积达到150万亩以上。加大扶贫开发力度,确保7.3万贫困人口当年脱贫。新增蓄水能力6000万方,新增农村通自来水人口41万人。搞好引黄灌区续建配套建设,提高引水能力,新增节水灌溉面积7万亩。完成18处乡镇卫生院的改造任务,年内100%行政村全部纳入乡村卫生一体化规范管理。扩大新型农村合作医疗试点,全面建立城乡医疗救助制度,积极争创卫生村镇。继续抓好农村公路改造,年内投资5.6亿元,新建、改造农村柏油路800公里。大力发展沼气、太阳能等新型能源,加强农村能源环保建设。完善动物疫病防治体系,力夺防控高致病性禽流感工作的全面胜利。从今年开始,每年创建一批新农村建设示范村,积极开展和谐家庭、和谐村居、和谐乡镇创建活动,加快农村公共文化设施建设,倡导科学健康、文明向上的生活方式。

　　(二)加快“工业兴市”步伐。以建设装备制造业基地和发展优势骨干企业群为目标,抓好十大产业链配套延伸,着力推进滨州经济开发区、邹平、滨城和各县园区、工业、服务项目建设,支持优势产业集群率先崛起,培植有实力的企业、有名气的产品迅速成长,扶持高新技术产业突破发展,确保规模以上企业新增200家以上,其中销售收入10亿元以上的企业达到23家,魏桥集团销售收入确保450亿元,力争突破500亿元。全市规模以上工业增加值达到400亿元,增长30%。加快实施名牌带动战略,年内争创中国名牌1个、山东名牌5个,山东省著名商标4-5件。把发展民营经济放在更加突出的战略位置,发展和完善中小企业担保机构,加强民营企业信用制度建设,力争全市信用民营企业达到300家以上。

　　(三)进一步完善公共基础设施。加快滨州铁路建设步伐,力争年内全线贯通,滨州火车站开工建设;滨太(德烟铁路滨州段)铁路规划启动;京滨、威乌高速投资11亿元,完成路基和桥梁主体工程;滨太(滨德)高速投资2.5亿元,完成部分路基土方和桥涵工程。搞好新一轮市区及各县城、乡镇总体规划,抓好两条城市带规划及全市产业布局规划。投资14.5亿元,搞好城区道路、桥梁及配套工程建设。抓好“四环五海”工程配套建设,开发完善七十二湖、三十六桥等特色景点,力争完成20座桥、30个湖的建设任务。抓好新立河、秦台干沟沿岸绿化,增加绿化面积60万平方米。完善配套城市水系,开工北海扩建工程,实施张肖堂干渠等河道整治工程,扩大水面83万平方米。加快老城区和城中村改造,开工建设10个经济适用住房小区。高度重视各县城、边远乡村、城市背街小巷的建设和整治,进一步巩固发展国家级卫生城市成果。

　　(四)突出抓好开放型经济。继续实施全方位招商引资,主攻日韩港台、拓展东南亚、突破欧美等国家和地区,全年合同利用外资达到4.5亿美元(新口径),增长12.5%,实际利用外资2.9亿美元(新口径),增长23%,其中利用外商直接投资1.7亿美元,增长82%。面对长三角、珠三角、天津滨海新区进行区域招商,力争实现400亿元人民币招商目标。积极参加国际经贸展谈活动,进出口实现27亿美元,增长21%。年内1—2家企业实现境内外上市,魏桥纺织实现增发配股,募集资金总额达到15—20亿元人民币。

　　(五)繁荣发展现代服务业。重点培植壮大骨干流通企业,加快发展新型流通方式和经营业态,着重抓好10个物流中心和10大物流企业的升级,积极引进国内外商贸物流企业落户滨州。完善滨州国际会展中心设施和功能,搞好与国内外知名会展机构的合作。办好中国通用航空产业博览会、中国国际家纺精品博览会暨高层论坛、中国国际龙舟比赛等重大节会和会展活动。抓好旅游业重点项目建设,着力提升“孙子故里、四环五海”两大品牌,丰富名胜、红色、生态、民俗、工业、农业旅游,确保旅游业各项指标取得新突破。加快发展金融业,优化金融生态环境,深化农村信用社改革,鼓励银行、保险等机构开发新的服务业务,辖区内本外币贷款余额确保增长20%以上,中小企业贷款增长翻一番。举办好第五次银企合作促进会,争取签约贷款合同、协议资金突破500亿元。

　　(六)大力实施“科教兴滨”战略。全面实施素质教育,提高教学质量。对农村义务教育阶段的贫困学生实行“两免一补”(免课本费、杂费,对贫困家庭的寄宿生生活进行补助),通过发放政府助学券的方式确保贫困家庭的子女上得起学。大力发展职业教育,招生1.5万人,在校生规模达到4.5万人,确保技术学院申办成功。组织实施科技富民、科技创业和科技成果引进转化工程项目300项以上,引进高科技成果100项以上、高科技人才60名以上。力争全年完成各类技术开发项目700项,达到国际国内先进水平和填补国内空白的重大项目60项以上,争创省级认定的企业技术中心1-2家,新认定省级高新技术企业10家以上、国家级新产品2个以上。

　　(七)积极推进区域经济良性互动。支持、促进经济强县率先发展、加快发展,充分发挥带动和辐射作用。集中帮扶和突破阳信、惠民两县,帮助欠发达县多上项目,上大项目。继续实施支持20个强乡镇、帮扶20个弱乡镇的“双20”工程,支持各县区因地制宜,发展特色经济,壮大优势产业,促进黄河南北县区优势互补、良性互动。加快以沾化、无棣为重点的北带开发步伐,新建沿海防潮堤50公里,力争完成护砌40公里。做好滨州港口的规划、设计、论证、立项工作,积极推进港口引堤建设。借助黄骅大港,启动鲁北渤海大桥建设,打开滨州海上通道。滨州距黄骅港只有600米之隔,架起跨海大桥,联接我市港航建设,节省投入、节省时间,可以真正实现借港兴滨,事半功倍,提前实现滨州人盼建港、早建港、建大港、早通航的多年愿望。目前,沧州、滨州两市政府已正式签定协议,可望3月底启动!同时,加快发展造船业,配套渔港建设。大力发展盐业和油盐化工产业,开发临港经济。加快甜高粱制乙醇加工项目建设,年内基地面积力争达到3万亩。

　　(八)全面深化各项改革。加快国企改革步伐,年内完成滨印集团、滨州毛纺总公司、滨州一棉等企业的后续改革,市属流通企业改制面达到70%以上。滨州活塞上半年完成股权分置改革,鲁北化工、华纺股份争取年内制定出股改方案并组织实施。合理划分各级政府财权、事权,深化部门预算、国库集中支付、政府采购和收支两条线改革,统筹兼顾,确保重点,集中财力办大事。深化土地使用制度改革,保障全市各项建设特别是招商引资项目及时用地。继续深入推进农村税费改革,重点抓好乡镇机构、农村义务教育、县乡财政体制、农村卫生体制等配套改革。

　　(九)着力建设资源节约型、环境友好型社会。大力推进能源、资源节约,以节能、节水、节材、节地为重点,建立用水“总量控制、定额管理、分类计价、累进加价”制度;实施最严格的土地保护政策,促进土地集约化利用;积极开发风力发电等可再生能源和新能源;加快发展循环经济,积极推行清洁生产。大力发展高效低耗型产业和产品,年底万元GDP能耗降到1.0吨标准煤,耗水量低于150吨,定期公布各县区单位生产总值、单位工业增加值能耗及污染物排放情况。在项目建设中优先考虑环保准入门槛,从源头上控制和治理污染。抓好小清河流域和六河流域污染防控工作,关停、搬迁、改造、提高“四环”之内重点污染项目;运用市场化的办法促进污染防治,形成“排污必须付费、治污可以赚钱”的环境资源价格机制,确保已建成的7家污水处理厂满负荷运营,新建的3家年底前试运行。搞好水土保持、盐碱地改良工作。积极争创国家环保模范城市,不断改善生态和人居环境。

　　(十)全力打造“和谐滨州”。落实企业最低工资制度,努力提高城乡居民特别是低收入者的收入水平。落实就业再就业的各项政策,多渠道开发就业岗位,新增就业3.3万人,新增农村劳动力转移5万人。不断提高城乡困难群众最低生活保障水平,坚决守住有饭吃、有衣穿、有房住的“三有”底线,逐步解决困难家庭上学难、看病难“两难”问题。进一步规范完善城乡低保,开展失地农民养老保险工作,各类优抚对象优待金按时足额发放。大力推进城镇养老、医疗、失业、工伤、生育保险,不断扩大增保面。加快敬老院建设步伐,年内实现集中供养70%的目标。加快城市社区建设,提高公共服务功能。搞好国防动员,加强民兵预备役建设,积极争创“双拥模范城”,增进军政军民团结。打好平抑第四次人口出生高峰攻坚战,全面完成年度人口与计划生育目标任务。扎实做好民族宗教工作,大力发展少数民族经济和各项事业。

　　各位代表!为进一步贯彻落实科学发展观,加快构建社会主义和谐社会,市政府确定,在去年圆满完成八项民心工程的基础上,今年再组织实施“十大民心工程”。(一)实行领导、部门、精力三集中,加大对惠民、阳信的帮扶力度,力争使这两个困难县尽快告别欠发达。(二)按照中央建设社会主义新农村的要求,结合滨州实际,动员全社会力量,规划、培育“生产发展、生活宽裕、乡风文明、村容整洁、管理民主”的示范村20个左右,为社会主义新农村建设树立样板。(三)按照建设资源节约型和环境友好型社会的基本要求,下决心解决城乡环境污染问题,使全市水和空气质量指标上升一个新的层次,让老百姓喝上干净水,呼吸新鲜空气。(四)就业是民生之本。积极实施就业再就业工程,千方百计开辟公益性岗位,解决好“4050”人员就业问题,使绝大部分符合就业条件的人员都能有班上、有活干、有饭吃。(五)继续加大投入,加大力度,搞好蓄水和吃水工程建设,奋战两年,实现村村通自来水,彻底解决城乡群众吃水难的问题。(六)积极推行城镇医疗保险和新型农村合作医疗制度,利用三年的时间,建立起比较完善的城乡医疗保障体制机制,解决老百姓看病难的问题。(七)进一步完善弱势群体救助机制,使全市城乡特困家庭也能基本过上一般家庭的生活,不求都能过得好,但求都能过得去。(八)建立特困家庭大学生救助机制,实行政府、社会、家庭共同负责,确保考入本科院校的每个学生都能顺利入学,安心就学,完成学业,成为社会有用人才。(九)建立健全失地农民保障机制,使失地农民失地不失业、不失收、不失保,老有所养、老有所医。(十)制定优惠政策,降低建设成本,加快经济适用房建设,使绝大部分工薪阶层都能住上比较适中的住宅,实现居者有其屋。

　　市政府确定的十件大事和十大民心工程,关系到全市经济社会发展的大局,关系到全市人民最关心、最直接、最现实的利益。加快发展是各级政府的首要任务,为人民服务是人民政府的神圣职责。市政府决心坚持以人为本、全面协调可持续的发展观,坚持发展为了人民,发展依靠人民,发展成果由人民共享,带着赶超跨越的激情,为滨州加快发展干大事、办难事;带着真挚深厚的感情,为人民群众办实事、做好事,把工作精力向十件大事和十大民心工程集中,分解目标,落实责任,脚踏实地,埋头苦干,向全市人民交一份满意的答卷!

　　五、依法行政,务实高效，促进政府自身建设再上新水平

　　面对新的形势和任务,各级政府要按照科学发展观和建设和谐社会的要求,努力做到“为民、务实、清廉”,全面提高行政能力和管理水平。

　　(一)着力提高依法行政的能力。以学习贯彻《公务员法》为契机,把依法行政贯彻到政府的各项工作中,切实做到有权必有责、用权受监督、侵权须赔偿、违法要追究。坚持向人大及其常委会报告工作和向人民政协通报情况制度,自觉接受人大及其常委会的依法监督,政协、各民主党派和人民团体的民主监督,广大人民群众和新闻舆论的监督。进一步完善基层民主管理制度,普遍推行村务、厂务和政务公开。加强司法监督,维护司法公正,支持审判机关和检察机关依法独立行使审判权和检察权。加强对基础设施建设、重点工程的审计监督,确保项目建设质量,提高政府投资效益。深入开展普法教育,做好法律服务和法律援助工作。4月份审批服务中心、市长公开电话受理中心、政府采购中心迁入新址,规范运行,县级行政审批服务中心6月底前全部建成,真正把各级审批服务中心建成为民、便民、利民、亲民的窗口。

　　(二)着力提高执政为民的能力。进一步加强作风建设,力戒官僚主义、形式主义,大兴求真务实之风。树立正确的政绩观,说实话、办实事、求实效,坚决防止和克服弄虚作假、浮夸虚报。加快建立为群众办实事、解难事的长效机制,根据群众需要和财力可能,从长远考虑、从眼前着手,从大事着眼、从具体事做起,每年排出若干事项,作出承诺,尽职尽责地抓紧抓好。各级政府公务员无论职位高低、权轻权重,都必须自觉为人民掌好权、用好权,领导干部要率先垂范,切实做到对党的事业高度负责,让人民群众放心满意!

　　(三)着力提高改革创新的能力。牢固树立市场经济理念,坚持用发展的办法解决前进中的问题。加大公益性事业推向市场的力度,广泛吸引社会资金加快经济社会发展。切实创新领导方式,把更多的精力转移到社会管理、公共服务上来。敢于突破前人,在“上级没有布置,前人没有做过,全国没有先例”的工作上闯新路、出新招,善于抓合乎规律的事,全力抓合乎国家法规和政策的事,主动抓合乎社情民意的事,以工作的创新推动各项事业的大发展。

　　(四)着力提高统筹落实的能力。事在人为,业在人创,发展在实干。站在全市经济社会发展的大局,在纷繁复杂的任务面前,统筹运作,分出轻重缓急,抓住关键环节,合理调配力量,科学组织实施。凡是确定的工作都要咬住不放,苦干实干,一抓到底,务求必胜。进一步完善决策目标、执行责任、考核监督“三个体系”,切实加强执法监察、效能监察,创新督查机制,确保各项工作有部署、有督查、见实效。

　　(五)着力提高维护安全稳定的能力。没有稳定,什么事情也干不成。按照“重心下移、强化基层、落实责任”的方针,全面加强“平安滨州”建设,不断提高管理社会事务、协调利益关系、处理人民内部矛盾的能力。加强安全监管,落实安全责任,杜绝重特大安全事故。加大市场和食品药品监管力度,确保市场秩序和食品、药品安全。依法及时合理解决人民群众反映的各类问题,加强社会治安综合治理和治安防控体系建设,严厉打击各种刑事犯罪。加强应急管理工作,形成反应灵敏、运转高效的应急机制,提高保障公共安全和处置突发事件的能力。

　　(六)着力提高廉洁勤政的能力。认真贯彻全市《惩治和预防腐败体系规划》及《实施细则》,坚持惩防并举、注重预防,从源头上治理腐败。加强政风行风建设,制定《全市政务公开考核办法》,以公开促廉政。从严治政,严肃法纪,着力解决经济管理部门和行政执法部门办事难、环节多、效率低的问题。进一步落实行政首长问责制和国家机关及公务员行政不作为问责办法,对不作为、乱作为的人和事,坚决查处,决不姑息!

　　各位代表!站在新起点,谋划新目标,开拓新思路,实现新发展,是历史赋予我们的神圣使命。让我们更加紧密地团结在以胡锦涛同志为总书记的党中央周围,高举邓小平理论和“三个代表”重要思想伟大旗帜,全面落实科学发展观,在市委的坚强领导下,以更大的气魄、更高的热情、更新的举措、更实的作风,再接再厉,乘胜前进,为建设民富市强的现代滨州而努力奋斗!
